# L-GSH Supplementation in Conjunction With Rifampicin Augments the Treatment Response to *Mycobacterium tuberculosis* in a Diabetic Mouse Model

**DOI:** 10.3389/fphar.2022.879729

**Published:** 2022-06-24

**Authors:** Abrianna Beever, Nala Kachour, James Owens, Kayvan Sasaninia, Afsal Kolloli, Ranjeet Kumar, Santhamani Ramasamy, Christina Sisliyan, Wael Khamas, Selvakumar Subbian, Vishwanath Venketaraman

**Affiliations:** ^1^ Graduate College of Biomedical Sciences, Western University of Health Sciences, Pomona, CA, United States; ^2^ College of Osteopathic Medicine of the Pacific, Western University of Health Sciences, Pomona, CA, United States; ^3^ Public Health Research Institute(PHRI) Center at New Jersey Medical School, Rutgers University, Newark, NJ, United States; ^4^ College of Veterinary Medicine, Western University of Health Sciences, Pomona, CA, United States

**Keywords:** diabetes, tuberculosis, host immune response, cytokine imbalance, redox imbalance

## Abstract

Both active tuberculosis (TB) and asymptomatic latent *Mycobacterium tuberculosis* (*M. tb*) infection (LTBI) cause significant health burdens to humans worldwide. Individuals with immunocompromising health conditions, such as Type 2 Diabetes Mellitus (T2DM), have a weakened ability to control *M. tb* infection and are more susceptible to reactivation of LTBI to active diseases. T2DM cases are known to have glutathione (GSH) deficiency and impaired immune cell function, including the granulomatous response to *M. tb* infection. We have previously reported that liposomal glutathione (L-GSH) supplementation can restore the immune cell effector responses of T2DM cases. However, the effects of L-GSH supplementation on the bactericidal activities of first-line anti-TB drug rifampicin (RIF) against *M. tb* infection have yet to be explored. The aim of this study is to elucidate the effects of L-GSH supplementation in conjunction with RIF treatment during an active *M. tb* infection in a diabetic mouse model. In this study, we evaluated total and reduced levels of GSH, cytokine profiles, malondialdehyde (MDA) levels, *M. tb* burden, and granulomatous response in the lungs. We show that L-GSH supplementation caused a significant reduction in *M. tb* burden in the lungs, decreased oxidative stress, and increased the production of IFN-γ, TNF-α, IL-17, IL-10, and TGF-β1compared to the untreated mice. In addition, L-GSH supplementation in conjunction with RIF treatment achieved better control of *M. tb* infection in the lungs and significantly reduced the levels of oxidative stress compared to treatment with RIF alone. Moreover, L-GSH in conjunction with RIF significantly increased TGF-β1 levels compared to treatment with RIF alone. These findings suggest potential therapeutic benefits of L-GSH supplementation in conjunction with first-line antibiotic therapy against *M. tb* infection in individuals with T2DM.

## Introduction

Tuberculosis (TB) accounts for one of the top 10 causes of mortality from a single infectious agent; roughly 1.5 million people died from this infection in 2020 (World Health Organization, 2021). The causative agent of TB, *Mycobacterium tuberculosis (M. tb),* is transmitted through aerosols. Inhalation of *M. tb* via aerosols can lead to active or latent TB infection primarily in the lungs; however, the bacteria can also infect distal organs if entered into the bloodstream. This disease is further fueled by the rapid spread of drug-resistant TB strains and weakened immune systems in the immunocompromised populations such as those afflicted with type 2 diabetes mellitus (T2DM).

During *M. tb* infection, alveolar macrophages play a pivotal role in providing the first line of defense against the pathogen (Rothchild et al., 2019). Pathogen-associated molecular patterns (PAMPs) on the mycobacterial cell envelope induce phagocytic uptake by the alveolar macrophages, triggering a T-cell mediated Th1 cytokine response. This response activates the pathways involved in producing cytokines, such as interleukin-2 (IL-2) and interferon-gamma (IFN-γ), to coordinate an immune response to prevent the growth and dissemination of *M. tb* (Allen et al., 2015; de Martino et al., 2019). A proper Th1 response will lead to the formation of a granuloma to contain the *M. tb* infection. Granulomas are complex and compact aggregates of immune cells that encapsulate infected cells at the site of infection. These immune cells include blood-derived macrophages, epithelioid cells, foamy macrophages, and multinucleated giant cells, both infected and non-infected with *M. tb* and surrounded by lymphocytes (Guirado and Schlesinger, 2013). Bacterial virulence factors in *M. tb* enable resistance to phagocytic degradation and allow the *mycobacterium* to remain dormant in granulomas, potentially for decades, leading to a latent TB infection (LTBI). Patients with LTBI are typically asymptomatic; however, patients with T2DM are at an increased risk of reactivation of LTBI due to increased risk of granuloma failure (Verma et al., 2021). Chronic inflammation and oxidative stress in T2DM lead to the accumulation of caseum due to necrosis in the lung parenchyma, which diminishes granuloma formation allowing for systemic *mycobacterium* dissemination and active infection 5. Active, cavitary disease is contagious and can lead to permanent lung disability due to tissue remodeling via healed cavitation, fibrosis, and bronchiectasis (Dheda et al., 2005). Investigating the pathways responsible for TB pathogenesis, especially in the context of co-morbidities, such as T2DM, and discovering methods to enhance immune responses within the immunocompromised populations are of desperate concern.

Treatments for *M. tb* infection involve a regimen of antibiotics, including isoniazid (INH), rifampicin (RIF), pyrazinamide (PZA), and ethambutol (ETH), lasting 6–9 months (Lagman et al., 2015). Due to the prolonged duration of treatment and severe side effects, the risk of patient non-compliance has increased, contributing to the development of multidrug-resistant TB (MDR-TB) (Ramappa and Aithal, 2013; Singh and Subbian, 2018). RIF is a current first-line antibiotic against *M. tb* infection (Campbell et al., 2001). RIF plays a role in anti-tuberculosis therapy by binding and inhibiting mycobacterial DNA-dependent RNA polymerase, disabling the bacteria’s ability to replicate (Wehrli, 1983; Campbell et al., 2001; Sharma et al., 2013). However, with the rise of MDR-TB, the dose is being re-evaluated to improve optimal bactericidal activity and decrease the length of treatment (Elliott et al., 1995; Goutelle et al., 2009; Maug et al., 2020). In order to combat *M. tb* treatment non-compliance and thus the surge in MDR-TB cases, it is imperative to explore additive therapies.

Increasing evidence suggests that Glutathione (GSH) serves an important role in the granulomatous response to *M. tb* infections. GSH is a tripeptide antioxidant composed of glutamate, cysteine, and glycine and is essential for maintaining host redox homeostasis. GSH consists of a reduced (rGSH) and oxidized (GSSG) form, with the functional form being rGSH, which prevents cellular damage from reactive oxygen species (ROS) by becoming oxidized by glutathione peroxidase, producing GSSG and water (Restrepo and Schlossberg, 2016). We have previously reported that GSH can have toxic effects on *M. tb* (Venketaraman et al., 2005; Venketaraman et al., 2008). Previous research has also found that liposomal glutathione (L-GSH) supplementation can improve the adaptive immune response against *M. tb* by maintaining CD4^+^ and CD8^+^ T cell viability and function within infected granulomas stimulating an increase in the production of the immuno-supportive cytokines IFN-γ and TNF-α and increasing autophagy (Abrahem et al., 2019). The induction of IFN-γ and TNF-α production allows for the formation of more robust granulomas and an enhancement of macrophage effector function to control the intracellular *M. tb* infection (Lin and Flynn, 2010).

Individuals with T2DM have been shown to have diminished levels of the GSH synthesis enzyme glutamine-cysteine ligase (GCLC), leading to lower levels of GSH. Along with diminished GSH, T2DM patients have an increase in levels of proinflammatory cytokines and MDA, a by-product of lipid peroxidation and measurement for oxidative stress (Guerra et al., 2012; Lagman et al., 2015; Teskey et al., 2018a). Previous studies have reported that when infected *in vitro* with *M. tb* Erdman strain, the peripheral blood mononuclear cells (PBMCs) from T2DM patients had impaired granuloma formation that is permissive for increased survival and growth of *M. tb,* compared to the PBMCs from healthy individuals (Teskey et al., 2018b; To et al., 2021). However, these effects were reversed when the infected cells were supplemented with L-GSH. Thus, supplementation of T2DM patients with L-GSH restored GSH levels, decreased levels of MDA, and enhanced the effector immune response to control the growth and survival of *M. tb in vitro* (Teskey et al., 2018b; Islamoglu et al., 2018; To et al., 2021). Previous *in vitro* studies have determined that restoring GSH levels in conjunction with treatment with anti-TB drugs, such as INH and RIF, in healthy individuals can enhance *M. tb* clearance in the granulomas generated with human PBMCs (Teskey et al., 2018b). Furthermore, macrophages isolated from T2DM individuals supplemented with GSH *in vitro* showed improved control of *M. tb* infection (Teskey et al., 2018b). These findings suggest that L-GSH, through its ability to modulate the immune response, can serve as adjunctive therapy, along with standard anti-TB drugs, for better control of *M. tb* growth and infection *in vitro*. However, the effects of L-GSH supplementation in conjunction with anti-TB drugs have yet to be elucidated *in vivo.*

Therefore, we hypothesized that L-GSH supplementation, in conjunction with RIF therapy, would improve the control *M. tb* infection in diabetic mice by regulating cytokine balance and diminishing oxidative stress of the host.

## Materials and Methods

### Bacteria and Chemicals

*Mycobacterium tuberculosis* H37Rv strain was cultured and prepared the stock for infection as described previously (Kolloli et al., 2021). Briefly, the bacteria were grown to OD600 = 0.6 to 0.8 in Middlebrook 7H9 medium (Difco BD, Franklin Lakes, NJ, United States) supplemented with 10% ADC (albumin dextrose catalase) enrichment (Difco BD, Franklin Lakes, NJ, United States). The *M. tb* culture was aliquoted and stored frozen at −80°C. The inoculum for infection was prepared by diluting stock vials as described previously (Tsenova et al., 2020). All chemicals were purchased from Millipore Sigma (Millipore Sigma, MA, United States) unless specified otherwise.

### Aerosol Infection of Mice, Treatment, and Bacterial CFU Assay

The diabetic C57BL *db/db* mice of 6–8 weeks old were purchased from Jackson Laboratories (Bar Harbor, ME, United States). *M. tb* inoculum for mice infection was prepared as described previously (Subbian et al., 2015). Mice were exposed to *M. tb* aerosols using Madison Chamber (Glas-Col LLC, Madison, WI, United States) optimized to deliver a standard low dose of about 100 CFU as reported previously (Cirillo et al., 2009). To estimate the actual bacterial numbers implanted in the lungs, three mice were sacrificed at 3 hours of post-infection (T = 0.). The lungs and spleen were harvested, and about 40% of the lungs were homogenized in 2 ml of sterile 1xPBS, serially diluted, and plated on Middlebrook 7H11 agar media (Difco BD, Franklin Lakes, NJ, United States). The number of bacterial CFUs was counted after 4-week incubation of the agar plates at 37°C with 5% CO2as described previously (Subbian et al., 2016a).

After infection, mice were randomly segregated into four groups: 1) no treatment, 2) treatment with 40 mM L-GSH, 3) treatment with RIF, and 4) treatment with a combination of RIF plus 40 mM L-GSH. All treatments were started on the day of infection and continued until the experimental endpoint (8 weeks post-infection). The L-GSH was supplied in drinking water and replaced every 3 days. RIF was administered at 16 mg/kg daily via oral gavage for the first 3 days. Since some *db/db* mice succumbed to this treatment probably due to RIF toxicity at 16 mg/kg, we reduced the dose to 8 mg/kg and treated the animals 3 times weekly through oral gavage from day-4 onwards until 8 weeks post-infection. At specific time points (4- and 8-weeks post-infection), three mice from each group were euthanized, and approximately 0.7 ml of blood was collected by cardiac puncture. A standard autopsy was performed, and lungs were harvested as described previously (Subbian et al., 2016a). The left lung (∼40% total weight) was homogenized in 2 ml sterile 1x PBS containing0.05% tween 80. The tissue homogenates were serially diluted and plated on Middlebrook 7H11 agar media. The number of bacterial CFUs was counted after 4-week incubation of the agar plates at 37°C with 5% CO_2_ as described previously (Subbian et al., 2016a).

**FIGURE 1:**
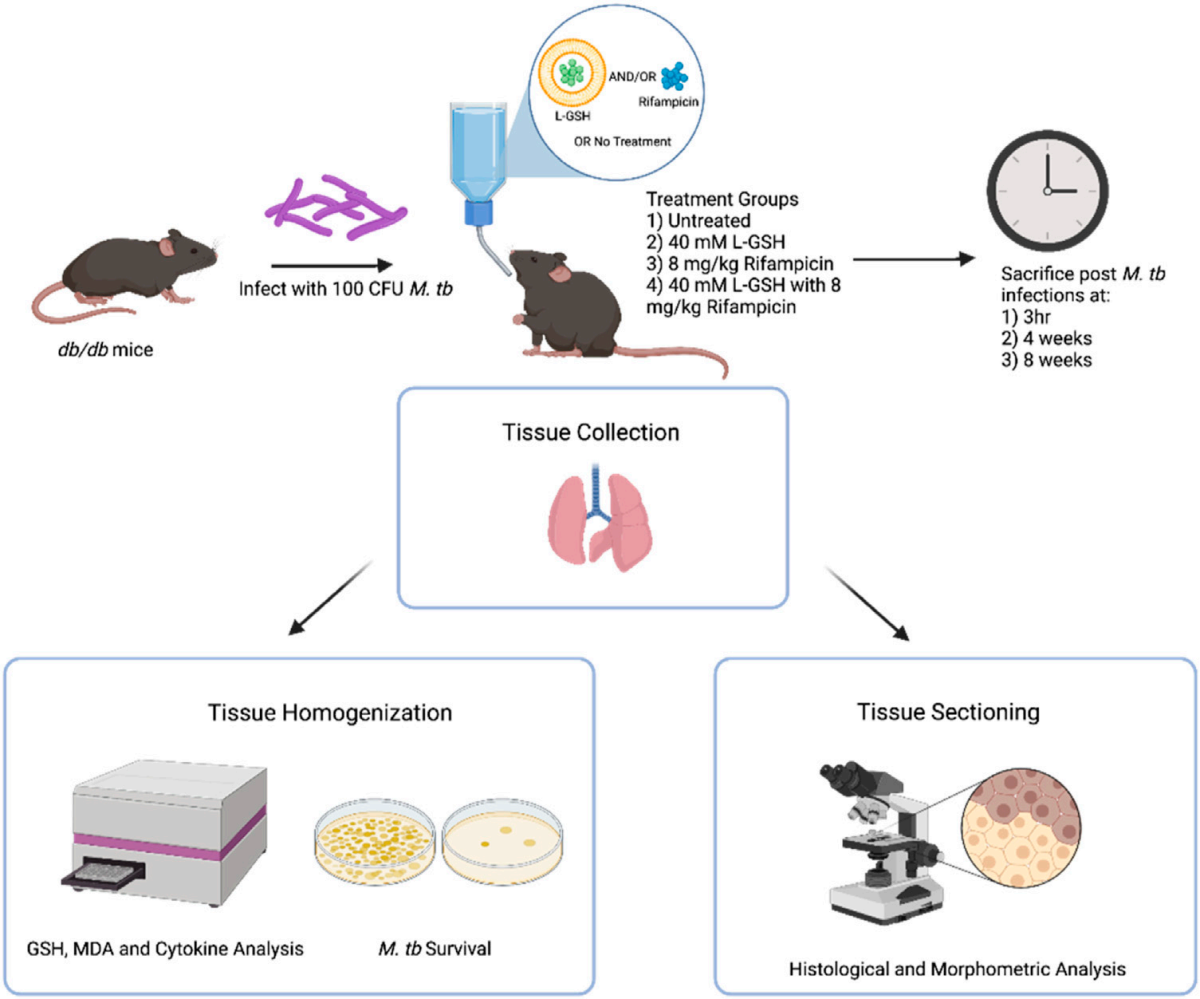
*Summary of experimental design*. Diabetic C57BL *db/db* mice were infected with 100 CFU *M tb* H37Rv. Mice were treated with 40 mM L-GSH, 8 mg/kg RIF, RIF +40mML-GSH, or untreated before sacrificing at 3 h (T = 0), 4-, and 8-weeks post-infection. Lung was collected and either homogenized for quantification of *M. tb* load as well as GSH, MDA, cytokine levels, and formalin-fixed for histological and morphometric analysis, respectively. Sample size (n) includes 3 mice per treatment and time category. This figure was created using biorender.com

For histology studies, the lower lobe of the right lung was fixed in 10% neutral buffered formalin. Lung lysates were filtered through a 0.2-micron filter and used for downstream analyses (Figure 1).

### Histology Staining of Lung Sections and Morphometry

Portions of the lungs fixed in 10% neutral formalin solution were paraffin-embedded, cut into 5 µm sections, and stained with Hematoxylin-Eosin (H&E) to visualize the organization of granulomas and distribution of leukocytes. The stained sections were analyzed using an Olympus Model BX41TF microscope and photographed using Olympus DP Controller.

For morphometric analysis of granuloma and immune cell infiltration, the H&E stained mice lung sections were analyzed using Path Scan Enabler-5 (Mayer Scientific, TX, United States) followed by morphometric determination of the area of lung involved in granulomatous response using Sigma Scan Pro (Systat Software Inc., CA, United States) as described previously (Subbian et al., 2016b).

### Quantification of Glutathione Levels

GSH levels were measured in the lung lysates of untreated, 40 mM L-GSH, RIF, and RIF +40 mM GSH treated diabetic mice at 4-weeks and 8-weeks post-infection. GSH levels were measured by using the Glutathione Colorimetric Detection Kit from Invitrogen (Cat. # EIAGSHC) following the manufacturer’s protocol (Thermofisher Scientific, Waltham, MA, United States). The rGSH (reduced GSH) was obtained by subtracting GSSG (oxidizedGSH) from the total GSH. All measurements were normalized to the total protein levels in the samples, and the results were reported in micromoles of GSH per Gram of protein.

### Quantification of Malondialdehyde Levels

MDA levels were determined in the lung tissue lysates of untreated, 40 mM L-GSH, RIF, and RIF +40 mM GSH treated diabetic mice at 4-weeks and 8-weeks post-infection. Lung lysates were measured spectrophotometrically using the Thiobarbituric Acid Reactive Substances (TBARS) assay kit procured from Cayman Chemicals (Cat # 10009055) following the manufacturer’s protocol (Cayman Chemicals, Ann Arbor, MI, United States). All measurements were normalized to the total protein levels in the samples, and the results were reported in micromoles of MDA per Gram of protein.

### Cytokine Measurement

Levels of IL-2, IL-6, IL-12, IL-17, IL-10, IFN-γ, TGF-β1, and TNF-α were measured in the lung tissue homogenates from untreated, 40 mM L-GSH, RIF, and RIF +40 mM L-GSH treated mice using enzyme-linked immunosorbent assay (ELISA) kits: IL-12 p70 Mouse Uncoated ELISA Kit (Cat # 88–7,121–88), IL-6 Mouse Uncoated ELISA Kit (Cat # 88–7,064–88), IL-2 Mouse Uncoated ELISA Kit (Cat #88–7,024–88), IL-17A (homodimer) Mouse Uncoated ELISA Kit (Cat # 88–7,371–86), IL-10 Mouse Uncoated ELISA Kit (Cat. # 88–7,105–88), IFN-γ Mouse Uncoated ELISA Kit (Cat. # 88–7,314–88), Human/Mouse TGF- β1 Uncoated ELISA Kit (Cat. # 88–8,350–88), TNF-α Mouse Uncoated ELISA Kit (Cat. # 88–7,324–88). All ELISA kits were procured from Thermofisher Scientific, and the cytokine levels were measured according to the manufacturer’s instructions (Thermofisher Scientific, Waltham, MA, United States).

### Quantification of Glucose Levels

Plasma glucose levels were determined via a colorimetric assay between untreated WT and *db/db M. tb-*infected mice 3 h post-infection. Additionally, plasma glucose levels of *M. tb-*infected *db/db* untreated, 40 mM L-GSH, RIF, and RIF +40 mM L-GSH treated mice were also assessed 4 and 8 weeks post-infection. The colorimetric assay was performed using a Glucose (GO) Assay Kit procured from Sigma-Aldrich (Cat #GAGO20) following the manufacturer’s protocol (Sigma-Aldrich, St Louis, MO, United States). Plasma glucose levels were measured with a spectrophotometer and reported in micrograms of glucose per milliliter of plasma.

### Statistical Analysis

For statistical analysis, GraphPad Prism Software 8 was utilized. One-way ANOVA with Brown-Forsythe and Welch correction was performed when comparing multiple groups. An unpaired T-test with Welch corrections was performed to determine the statistical significance between two groups. All values reported represent the means and standard deviations for each category, with *p*-values of <0.05 considered statistically significant. Any placement of an asterisk (*) denotes a direct comparison to the previous category. When two asterisks are represented (**), a *p*-value below 0.01 is implied. When three asterisks are represented (***), a *p*-value <0.005 is implied. When four asterisks are represented (****), a *p*-value <0.0001 is implied.

## Results

### Liposomal Glutathione Plus Rifampicin Treatment Reduced Malondialdehyde Levels and Increased Total Glutathione and Reduced Glutathione Levels in the Lungs of Diabetic Mice Infected With *M. tb*

**FIGURE 2:**
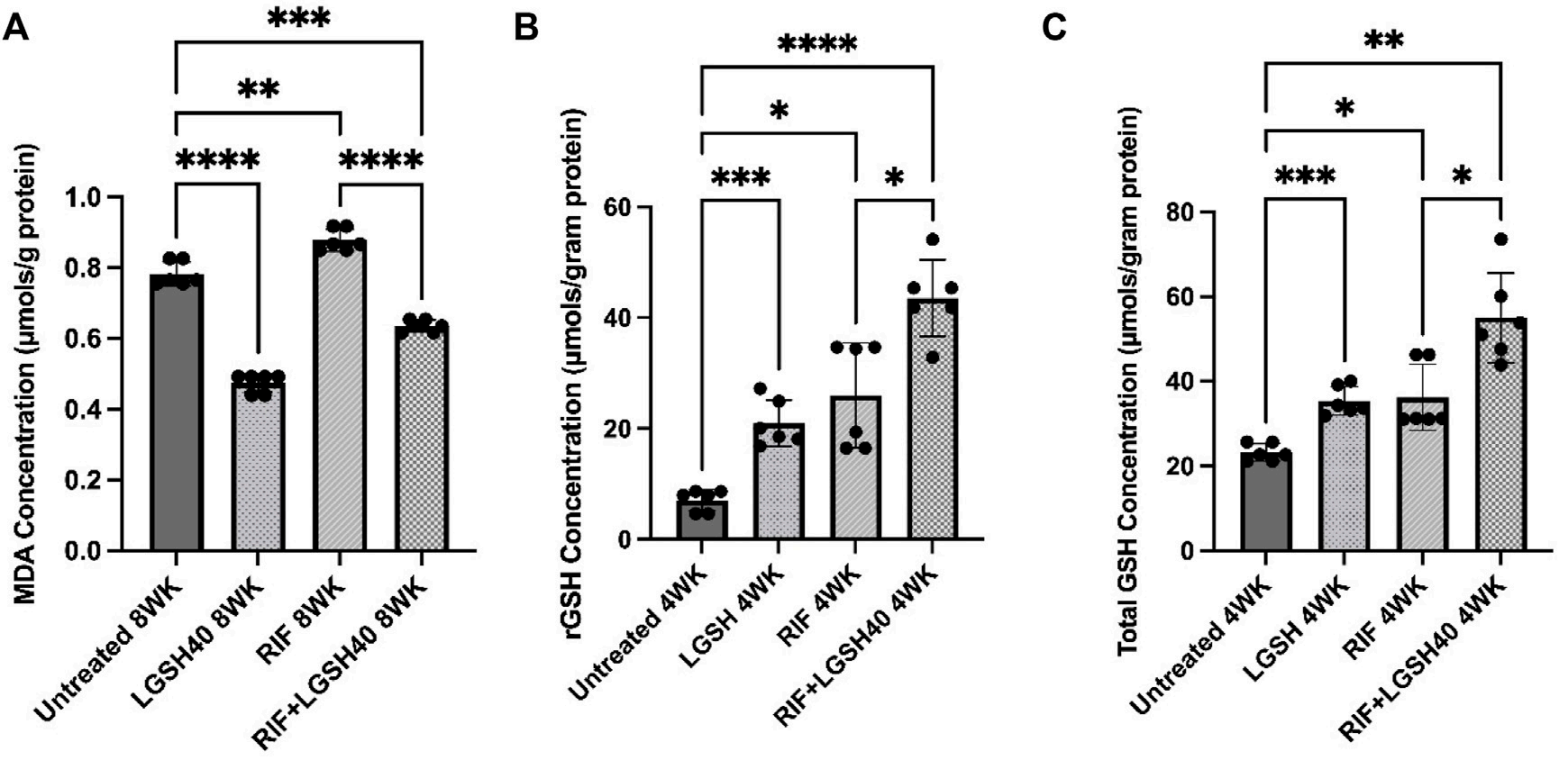
MDA,rGSH, and Total GSH measurements in the lung homogenates of untreated, RIF, L-GSH40, and RIF + L-GSH40 treated *M. tb* infected diabetic mice. MDA **(A)**, rGSH **(B)**, Total GSH **(C)** levels were measured spectrophotometrically. Lung lysates were collected from untreated, L-GSH40, RIF, and RIF + L-GSH treated mice at 4- and 8-weeks post *M. tb* infection. Statistical analysis was performed using GraphPad Prism Software 8. Values plotted represent the mean ± standard deviation for each group with n = 3 per group per time point. Data were analyzed as multiple groups using One-way ANOVA with Brown-Forsythe and Welch correction for unequal data dispersion. *p*-values of <0.05 (*), <0.01 (**), <0.005 (***), <0.0001 (****) were considered significant. Sample size (n) includes three mice/group in the 4-weeks and 8-weeks post-infection.

MDA levels were measured in the lung homogenates to determine the extent of oxidative stress in the *M. tb* infected *db/db* mice at the local (lungs) sites of infection. In comparison to the untreated *M. tb* infected *db/db* mice, L-GSH treatment caused a significant reduction in the levels of MDA in the lungs (Figure 2A). In contrast, RIF treatment caused a significant increase in MDA levels in the lungs compared to the untreated group (Figure 2A). Similarly, adjunctive treatment of L-GSH with RIF resulted in a significant reduction in MDA levels in the lungs compared to the untreated group and RIF-alone treatment group (Figure 2A). These results indicate that administration of L-GSH with and without RIF can alleviate oxidative stress induced by *M. tb* infection and/or RIF monotherapy.

Levels of rGSH, the antioxidant form of GSH, were measured in the lung tissues of *M. tb* infected *db/db* mice that were untreated, RIF, 40 mM L-GSH, or RIF + L-GSH treated. Compared to the untreated *M. tb* infected mice, L-GSH treatment resulted in a significant increase in the lung rGSHlevels (Figure 2B). Increased levels of rGSH correlate with the significantly decreased levels of the oxidative stress marker, MDA (Figure 2A). There was a significant increase in levels of rGSH in RIF-treated mice compared to the untreated *M. tb* infected *db/db* mice (Figure 2B). More importantly, there was a significant increase in rGSHlevels in *M. tb* infected *db/db* mice treated with a combination of RIF and L-GSH compared to the untreated mice with or without RIF treatment (Figure 2B). These results show that L-GSH in conjunction with RIF treatment, can significantly increase the levels of rGSH and reduce oxidative stress in the lungs of *M. tb* infected *db/db* mice.

Levels of the total form of GSH were measured in the lung tissues of *M. tb* infected *db/db* mice that were untreated, RIF, 40 mM L-GSH, or RIF + L-GSH treated. Treatment of *M. tb* infected diabetic mice with L-GSH resulted in a significant increase in total GSH levels compared to the untreated mice (Figure 2C). There was also a significant increase in total GSH levels in mice treated with RIF (Figure 2C). Most importantly, when compared to untreated mice and RIF alone treated mice, there was a significant increase in total GSH levels with a combination of RIF and L-GSH treatment (Figure 2C). These results correlate with levels of rGSH (Figure 2B).

### Liposomal Glutathione Plus RIF Treatment Increased IFN-γ and IL-12 Production in the Lungs

**FIGURE 3:**
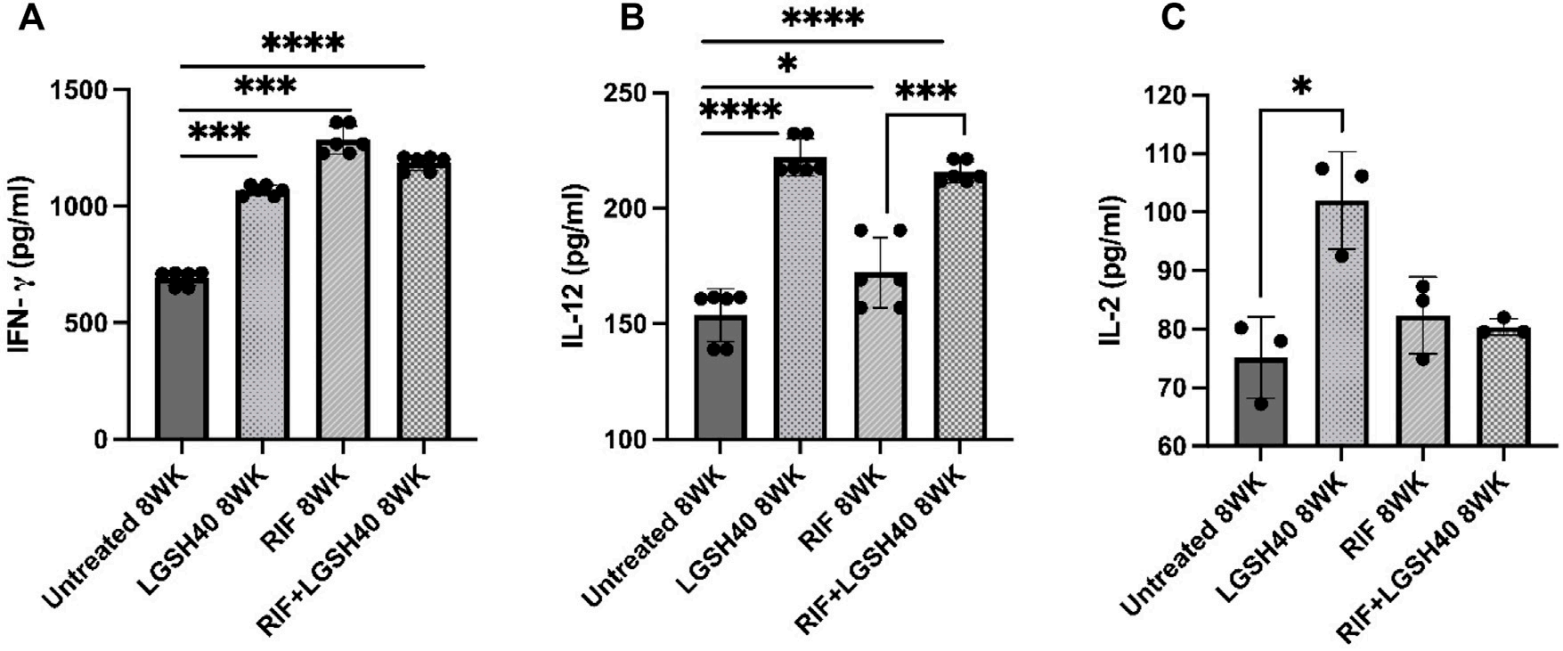
*IFN-γ, IL-12, IL-2 measurements in the lung homogenates of untreated, RIF, L-GSH40, and RIF + L-GSH40 treated M. tb infected diabetic mice*. IFN-γ **(A)**, IL-12 **(B)**, IL-2 **(C)** were measured spectrophotometrically. Lung lysates were collected from untreated, L-GSH40, RIF, and RIF + L-GSH treated mice at 8 weeks post *M. tb* infection. Statistical analysis was performed using GraphPad Prism Software 8. Values plotted represent the mean ± standard deviation for each group with n = 3 per group per time point. Data were analyzed as multiple groups using One-way ANOVA with Brown-Forsythe and Welch correction for unequal data dispersion. *p*-values of <0.05 (*), <0.01 (**), <0.005 (***), <0.0001 (****) were considered significant.

To elucidate the Th1 response in *M. tb* infected diabetic mice treated with L-GSH + RIF, we assessed the levels of Th1 cytokines; IFN- γ, IL-2, and IL-12. IFN-γ is produced by a Th1 subset of CD4^+^ T cells and enhances the antimycobacterial effector functions of macrophages, NK, and CD8 T cells. Our results show that when compared to untreated mice, treatment of *M. tb*-infected *db/db* mice with L-GSH alone and a combination of L-GSH and RIF resulted in a significant increase in the levels of IFN-γ in the lungs (Figure 3A). Treatment of *M. tb*-infected *db/db* mice with RIF alone also resulted in a significant increase in the levels of IFN-γ in the lungs (Figure 3A). Treatment of *M. tb*-infected mice with a combination of L-GSH and RIF resulted in a localized increase in the lung IFN-γ levels, which positively correlated with superior control of *M. tb* infection.

IL-12 is a polarizing cytokine that induces the differentiation of CD4 T-cells to the Th1 subset that produces IFN-γ and IL-2. Treatment of *M. tb*-infected *db/db* mice with L-GSH alone or a combination of L-GSH and RIF treatment resulted in a significant increase in IL-12 in the lungs compared to untreated mice (Figure 3B). The increase in IL-12levels correlated with the increase in lung IFN-γ levels of RIF + L-GSH treated *M. tb* infected *db/db* mice (Figure 3B). Treatment of *M. tb* infected *db/db* mice with RIF alone also resulted in a significant increase in IL-12 levels in the lungs (Figure 3B). There was also a significant increase in IL-12 levels in the lungs of RIF + L-GSH treated mice compared to RIF alone treatment (Figure 3B).

IL-2 is another cytokine produced by the Th1 subset of CD4^+^ T cells. IL-2 is a T cell growth factor that maintains T cell viability and amplifies T cell responses. When compared to untreated *M. tb* infected *db/db* mice, treatment with L-GSH alone resulted in a significant increase in IL-2 levels in the lungs (Figure 3C). However, treatment of *M. tb*-infected *db/db* mice with RIF alone and RIF + L-GSH did not increase IL- 2 levels (Figure 3C).

Overall, these results indicate that L-GSH in conjunction with RIF treatment, can significantly increase IL-12 and IFN-γ and help enhance the antimycobacterial functions of immune cells to combat *M. tb* infection.

### Liposomal Glutathione Plus Rifampicin Treatment Increased TGF-β1 and IL-10 Levels in the Lungs

**FIGURE 4:**
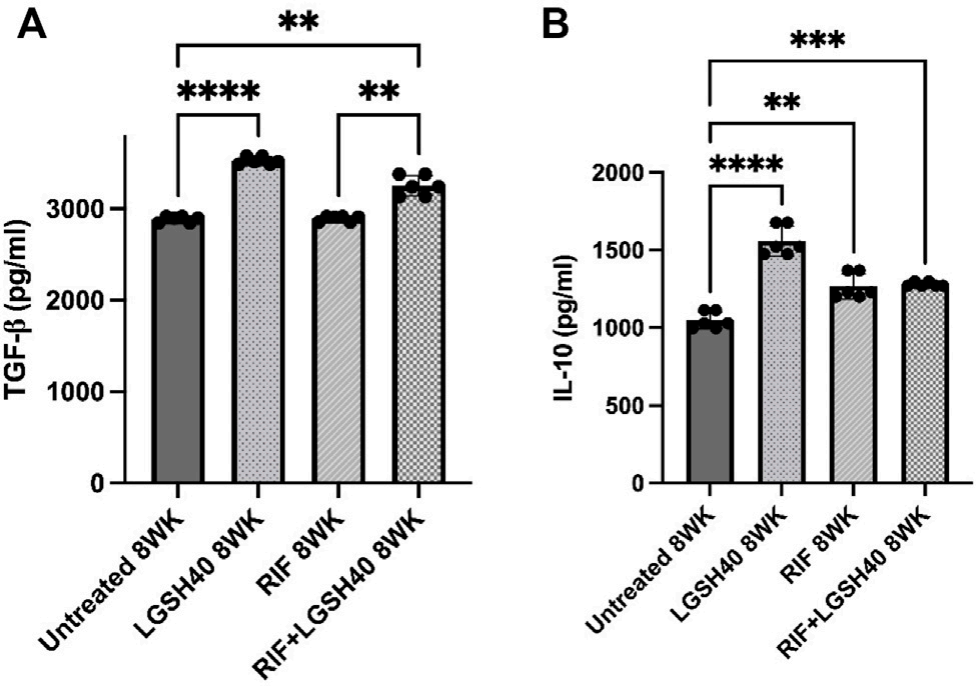
*TGF-*β1 *and IL-10 measurements in the lung homogenates of untreated, RIF, L-GSH40, and RIF + L-GSH40 treated M. tb infected diabetic mice.* TGF-β1 **(A)** and IL-10 **(B)** levels were measured spectrophotometrically. Lung homogenates were collected from untreated, L-GSH40, RIF, and RIF + L-GSH treated mice at 8 weeks post *M. tb* infection. Statistical analysis was performed using GraphPad Prism Software 8. Values plotted represent the mean ± standard deviation for each group with n = 3 per group per time point. Data were analyzed as multiple groups using One-way ANOVA with Brown-Forsythe and Welch correction for unequal data dispersion. *p*-values of <0.05 (*), <0.01 (**), <0.005 (***), <0.0001 (****) were considered significant.

Levels of TGF-β1 and IL-10 were measured in *M. tb* infected diabetic miceto assess the effects of L-GSH + RIF treatment in altering the production of these cytokines. TGF-β1 produced by macrophages and regulatory T cells is also an immunomodulatory and anti-inflammatory cytokine. Our results show that treatment of *M. tb*-infected *db/db* mice with L-GSH resulted in a significant increase in TGF-β1 levels in the lungs compared to untreated mice (Figure 4A). Furthermore, compared to the untreated *M. tb* infected *db/db* mice, treatment with a combination of L-GSH and RIF also resulted in a significant increase in TGF-β1 levels (Figure 4A). Additionally, treatment of *M. tb*-infected *db/db* mice with RIF alone did not show any significant rise in TGF-β1 levels (Figure 4A). Treatment of *M. tb*-infected *db/db* mice with L-GSH alone or L-GSH in conjunction with RIF resulted in a significant increase in TGF-β1 levels (Figure 4A). These results further signify the effects of L-GSH in decreasing the burden of *M. tb* burden and restoring homeostasis in the immune responses in the lungs by upregulating TGF-β1production.

Additionally, IL-10 is also an anti-inflammatory and immunomodulatory cytokine. When compared to the untreated *M. tb* infected *db/db* mice, treatment with L-GSH in the presence and absence of RIF resulted in a significant increase in IL-10 levels in the lungs (Figure 4B). Treatment of *M. tb*-infected *db/db* mice with RIF alone also resulted in a significant increase in the levels of IL-10 (Figure 4B). Overall, the results show that treatment of *M. tb*-infected *db/db* mice with L-GSH alone, RIF-alone and L-GSH + RIF resulted in a significant increase in the levels of IL-10. These results indicate that by reducing the *M. tb* burden, L- GSH and RIF can modulate the inflammatory immune responses in the lungs by inducing IL-10 production.

### Liposomal Glutathione Treatment Enhanced IL-17 Levels in the Lungs

**FIGURE 5:**
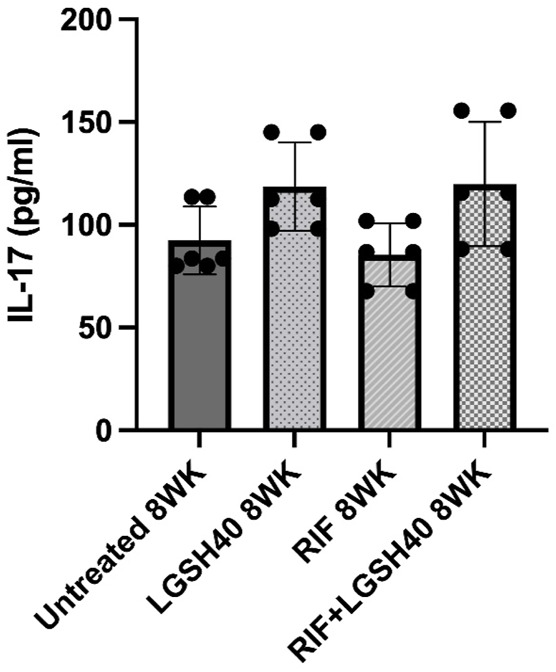
IL-17 measurements in the lung homogenates of untreated, RIF, L-GSH40, and RIF + L-GSH40 treated M. tb infected diabetic mice. IL-17 levels were measured spectrophotometrically. Lung lysates were collected from untreated, L-GSH40, RIF, and RIF + L-GSH treated mice at 8 weeks post *M. tb* infection. Statistical analysis was performed using GraphPad Prism Software 8. Values plotted represent the mean ± standard deviation for each group with n = 3 per group per time point. Data were analyzed as multiple groups using One-way ANOVA with Brown-Forsythe and Welch correction for unequal data dispersion. *p*-values of <0.05 (*), <0.01 (**), <0.005 (***) were considered significant.

IL-17, produced by the Th17 subset of CD4 T cells, activates the immune effector mechanism to control *M. tb* infection. Our results show that L-GSH treatment caused an insignificant increase in IL-17 levels in the lungs compared to untreated *M. tb* infected *db/db* mice (Figure 5). No changes in the levels of IL-17 were observed in the RIF-alone treated animals (Figure 5B). However, treatment of *M. tb* infected *db/db* mice with a combination of L- GSH and RIF resulted in a noticeableincrease in IL-17 levels in the lungs compared to the RIF-alone treatment group, however, this was insignificant (Figure 5). Overall, treatment with a combination of RIF and L-GSH resulted in a noticeable but insignificant increase in IL-17 levels in the lungs, indicating that GSH could augment immune effector functions by elevating IL-17.

### Liposomal Glutathione Treatment Modulates TNF-α and IL-6 Production in the Lungs

**FIGURE 6:**
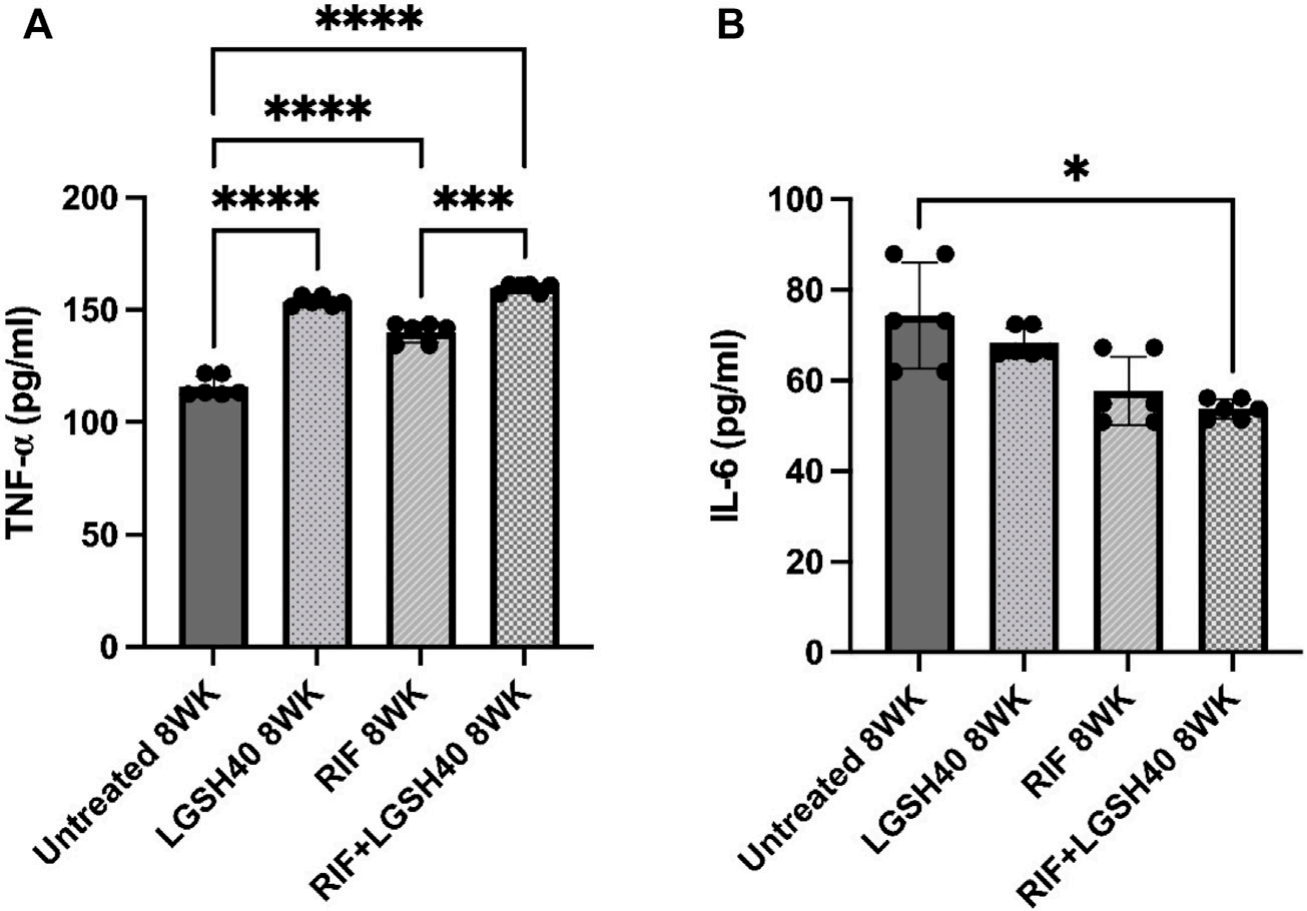
*TNF-*α *and IL-6 measurements in the lung lysates of untreated, RIF, L-GSH40, and RIF + L-GSH40 treated M. tb infected diabetic mice.* TNF-α **(A)** and IL-6 **(B)** levels were measured spectrophotometrically. Lung lysates were collected from untreated, L-GSH40, RIF, and RIF + L-GSH treated mice at 8 weeks post *M. tb* infection. Statistical analysis was performed using GraphPad Prism Software 8. Values plotted represent the mean ± standard deviation for each group with n = 3 per group per time point. Data were analyzed as multiple groups using One-way ANOVA with Brown-Forsythe and Welch correction for unequal data dispersion. *p*-values of <0.05 (*), <0.01 (**), <0.005 (***) were considered significant. Sample size (n) includes three mice/group.

TNF-α is essential for granuloma formation and maintenance; it activates macrophage effector functions. Treatment of *M. tb*-infected *db/db* mice with L-GSH with or without adjunctiveRIF resulted in a significant increase in TNF-α levels compared to the untreated mice (Figure 6A). Similarly, compared to the untreated *M. tb*-infected *db/db* mice, treatment with RIF alone also significantly increased the levels of TNF-α (Figure 6A). These results suggest that the elevated TNF-α levels of in *M. tb*-infected *db/db* mice upon treatment with L-GSH, RIF or a combination of L-GSH + RIF can help the granulomatous response in the lungs.

IL-6, a pro-inflammatory cytokine, is implicated in the induction of oxidative stress. In comparison to the untreated *M. tb*-infected *db/db* mice, treatment with L-GSH alone resulted in a modest decrease in IL-6 levels (Figure 6B). However, treatment of *M. tb*-infected *db/db* mice with RIF alone resulted in a noticeable but insignificant decrease in IL-6 levels and a significant reduction in mice treated with RIF + L-GSH compared to untreated mice (Figure 6B).

### Rifampicin Plus Liposomal Glutathione Treatment Reduces *M. Tb* Burden in Mice Lungs

**FIGURE 7:**
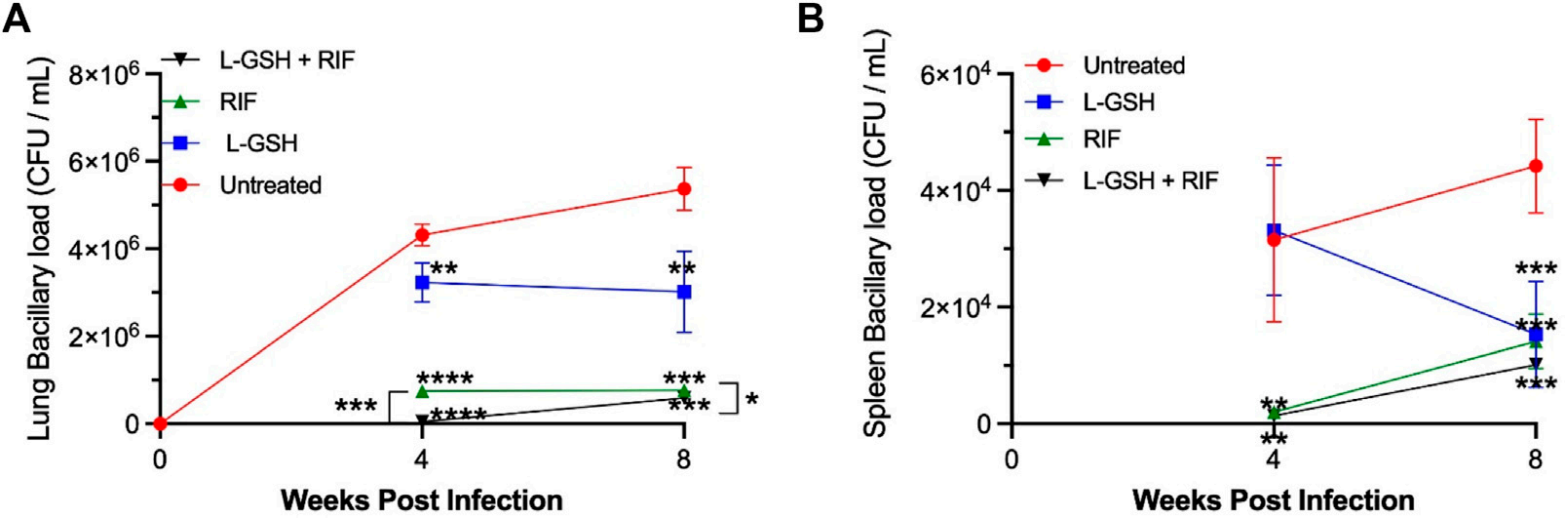
*M. tb* burden in the lungs and spleen of untreated, 40 mM L-GSH, RIF, and RIF + L-GSH treated diabetic mice at 4-weeks and 8-weeks post-infection. *M. tb* survival in the lung was quantified via CFU assays performed on lung homogenates of *M. tb*-infected diabetic mice untreated and treated with 40 mM L-GSH, RIF, or RIF + L-GSH treatment at 4 weeksand 8 weeks post-infection **(A)**. *M. tb* survival in the spleen was quantified via CFU assays performed on the spleen homogenates of *M. tb*-infected diabetic mice untreated and treated with 40 mM L-GSH, RIF, or RIF + L-GSH treatment at 4 and 8 weeks post-infection **(B)**. GraphPad Prism Software 8 was utilized for statistical analysis. Values plotted represent the mean ± standard deviation for each group with n = 3 per group per time point. Data were analyzed as multiple groups using One-way ANOVA with Brown-Forsythe and Welch correction for unequal data dispersion. *p*-values of <0.05 (*), <0.01 (**), <0.005 (***) were considered significant. Asterisks indicate difference from untreated mice unless otherwise noted with brackets.

To determine the ability of diabetic mice to contain bacterial survival, *M. tb* load in the lung was determined by CFU assay. A baseline was established by comparing all groups to the untreated *M. tb* infected *db/db* mice. L-GSH treatment caused a significant reduction in *M. tb* burden in the lungs at 4- and 8-weeks post-infection, compared to the no-treatment group (Figure 7A). Treatment with RIF-alone also resulted in a more significant (>75%) reduction in *M. tb* survival in the lungs at 4- and 8-weeks post-infection. Importantly, when *M. tb* infected *db/db* mice were treated with a combination of RIF plus L-GSH, a highly significant (>90%) reduction in lung bacterial load was noted at 4- and 8-weeks post-infection, compared to the no-treatment group (Figure 7A).

### Liposomal Glutathione Treatment Reduces *M. Tb* Burden in Mice Spleen

*M. tb* load in the spleen of diabetic mice was determined by CFU assay. In comparison to the untreated *M. tb* infected diabetic mice, L-GSH treatment caused a reduction in the *M. tb* burden in the spleen at 4-weeks post-infection, however this was not significant until 8-weeks post-infection where there was a 60% decrease in the viability of *M. tb* in the spleen at 8-weeks post-infection (Figure 7B). Treatment with Rifampicin resulted in a drastic reduction in the viability of *M. tb* in the spleen at 4 and 8-weeks post-infection (Figure 7B). Treatment with a combination of L-GSH and rifampicin caused a further noticeable decrease in the viability of *M. tb* in the spleen. However, this decrease was not significant at 4- or 8-weeks post-infection compared to RIF treatment alone (Figure 7B).

### Rifampicin and Rifampicin + Liposomal Glutathione Treatment Reduced *M. Tb* Burden in Mice Liver

*M. tb* burden in the liver of diabetic mice was determined by CFU assay. Compared to the untreated *M. tb* infected diabetic mice, L-GSH treatment did not result in a significant reduction in *M. tb* survival in the liver at 4-weeks post-infection (Supplemental Figure S3). Treatment with rifampicin resulted in a log decrease in the number of *M. tb* in the liver at 4 weeks post-infection (Supplemental Figure S3). Treatment with a combination of L-GSH and rifampicin also caused a significant diminishment in the viability of *M. tb* in the liver (Supplemental Figure S3).

*Treatment with RIF plus L-GSH reduces granulomatous response in M. tb infected mice lungs.* To determine the effect of RIF + L-GSH treatment on the lung pathology and granulomatous response of *M. tb* infected *db/db* mice, we performed a histopathologic analysis of lung sections and morphometric measurement of granulomas at 4- and 8-weeks post-infection.

**FIGURE 8:**
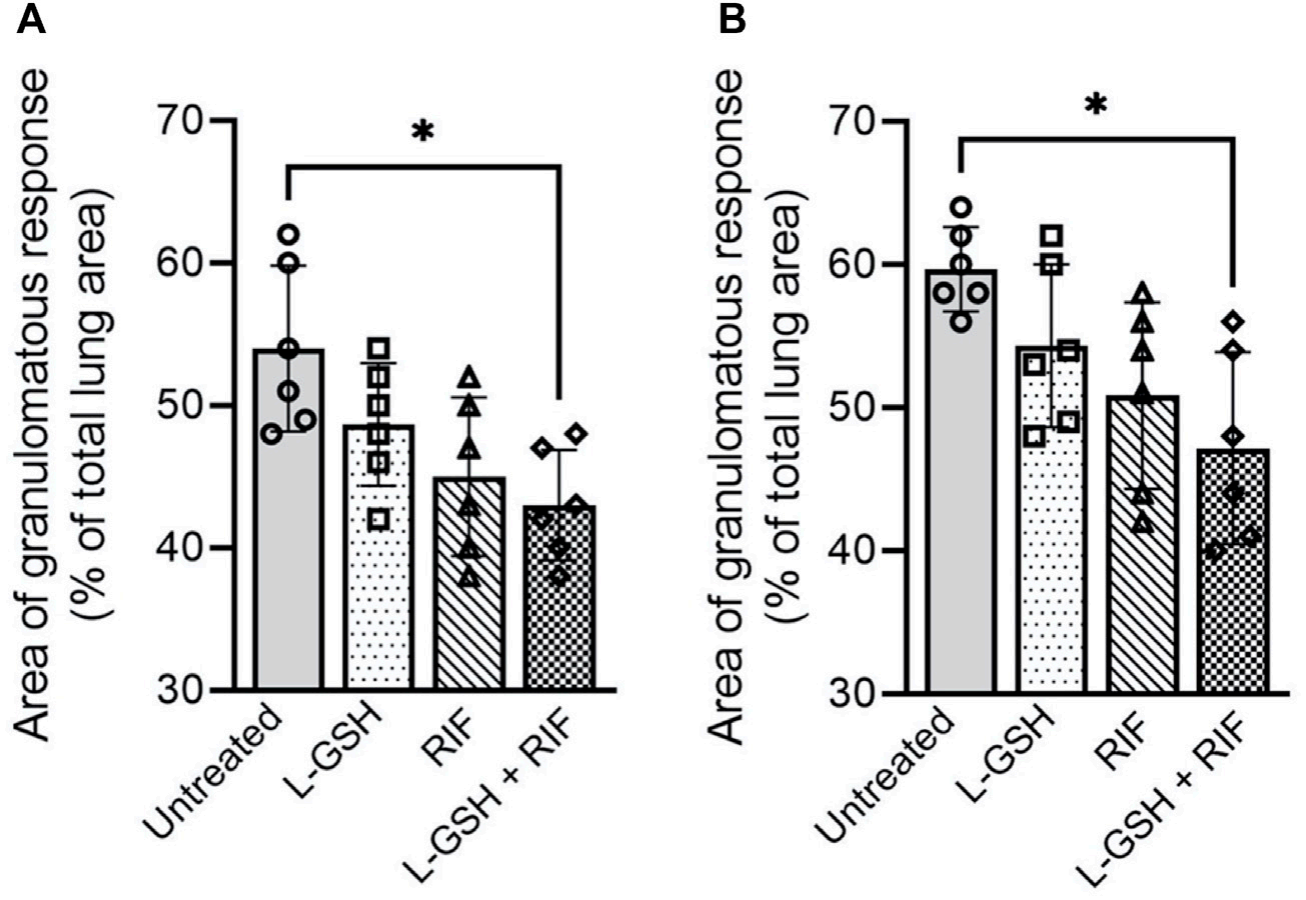
*Morphometric analysis of lung pathology in M. tb infected db/db mice.* The area of lung involved in granuloma formation at 4 weeks **(A)** and 8 weeks **(B)** post-infection were determined by morphometry and presented as a percentage of the total lung area. L-GSH was treated at 40mM, and RIF was treated at 16 mg/kg. Values plotted represent the mean ± standard deviation for each group with n = 3 per group per time point. Data were analyzed as multiple groups using One-way ANOVA with Brown-Forsythe and Welch correction for unequal data dispersion. *p < 0.05 compared to the untreated group.

At 4 weeks post-infection, an average of about 55% of the lungs were involved in the granulomatous response in the untreated *M. tb*infected*db/db* mice, compared to an average lung involvement of about 49% in 40 mM L-GSH-alone treated mice and about 45% in RIF-alone treated mice. However, the difference was not statistically significant between these groups (Figure 8A). In contrast, a significant reduction in the granulomatous response was noted in *M. tb* infected *db/db* mice treated with a combination of RIF + L-GSH, compared to the untreated group (∼43 versus ∼55%). At 8 weeks post-infection, the percent of lung involvement in granuloma formation of the untreated mice increased to an average of about 60% (Figure 8B). Although the lung area involved in granuloma formation was reduced in the L-GSH-alone treated and RIF-alone treated mice, the difference was not statistically significant compared to the untreated animals. However, similar to the results observed at 4 weeks post-infection, a significant reduction in the granulomatous response was noted at 8 weeks post-infection in *M. tb* infected *db/db* mice treated with a combination of RIF + L-GSH, compared to the untreated group (∼47 versus ∼60%).

**FIGURE 9:**
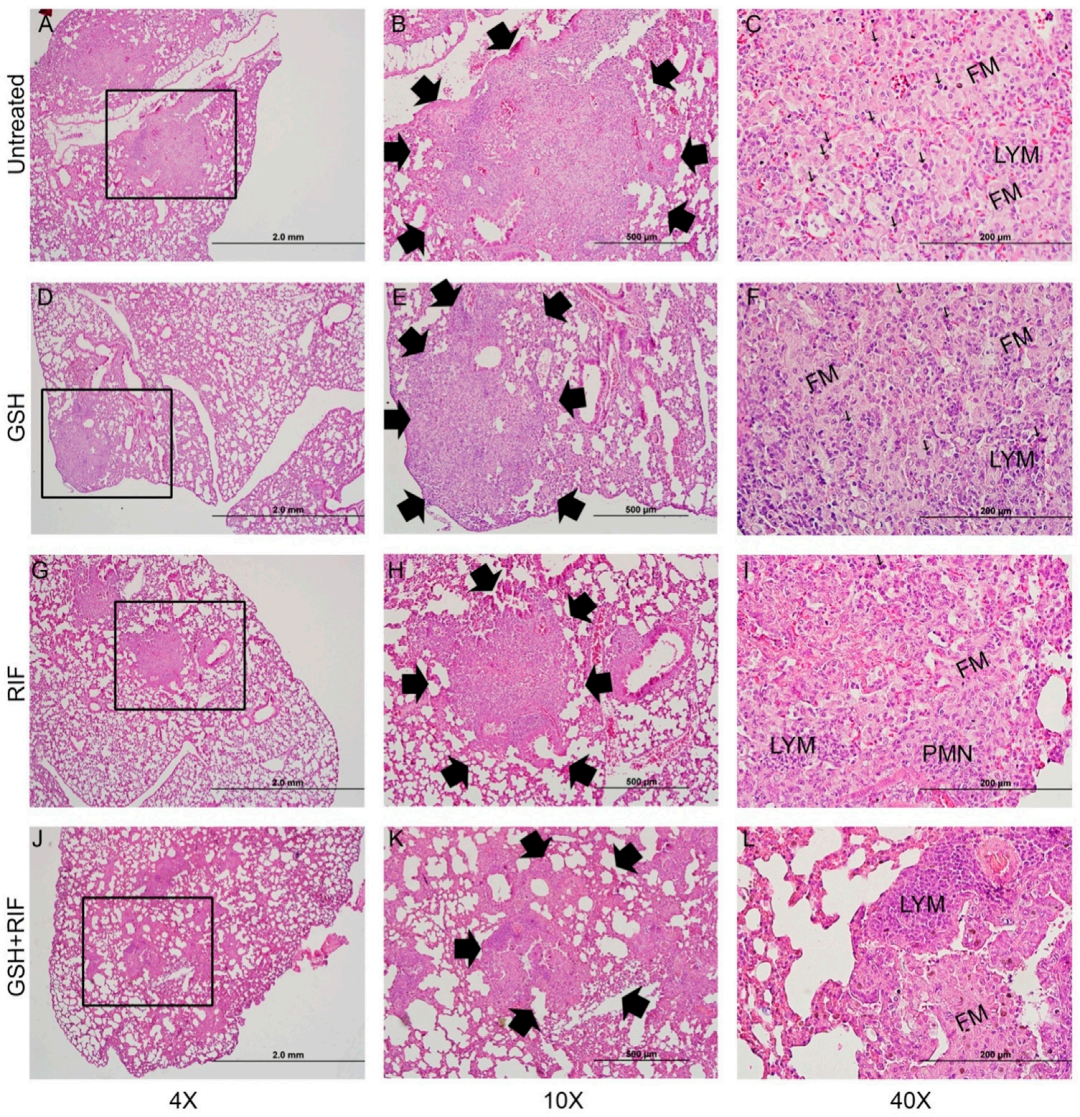
*Histology of M. tb infected db/db mice at 4 weeks post-infection.* Representative lung sections were collected from *M. tb* infected *db/db* mice with or without various treatments at 4-weeks post-infection for H&E staining. Animals were either untreated **(A–C)** or treated with L-GSH (GSH; **(D–F)** or RIF **(G–I)** or a combination of L-GSH plus RIF (GSH + RIF; J-L). Images were photographed at 4X **(A,D,G,J)** or 10X **(B,E,H,K)** or 40x **(C, F, I, L)** magnifications. Boxes in 4X magnification represent the granulomatous region magnified in 10X. Arrows in 10X magnification show the boundary of granulomas. Arrows in 40X show neutrophils (polymorphonuclear cells; PMN). FM-foamy macrophages; LYM-lymphocytes. The scale bar in 4X is 2 mm; 10X is 500µm and 40X is 200 µm.

The lung histopathologic analysis at 4 weeks post-infection revealed multiple and poorly demarcated granulomatous lesions in all *M. tb* infected *db/db* mice, irrespective of any treatment (Figure 9A–L). Abundant foamy macrophages interspersed with lymphocytes were seen in all these granulomas. No obvious central necrosis was noted in any of the granulomas. Consistent with the morphometric analysis, multiple, large granulomas with a prominent influx of polymorphonuclear neutrophils (PMN) were noted in the untreated mice lungs (Figures 9A–C), compared to the various treatment groups. Mice treated with L-GSH-alone (Figures 9D–F) or RIF (Figures 9G–I) alone had multiple, smaller granulomas with minimal PMN influx than the untreated group. However, the granulomas were much reduced in size and numbers in the lungs of mice treated with a combination of RIF + L-GSH (Figures 9J–L).

**FIGURE 10:**
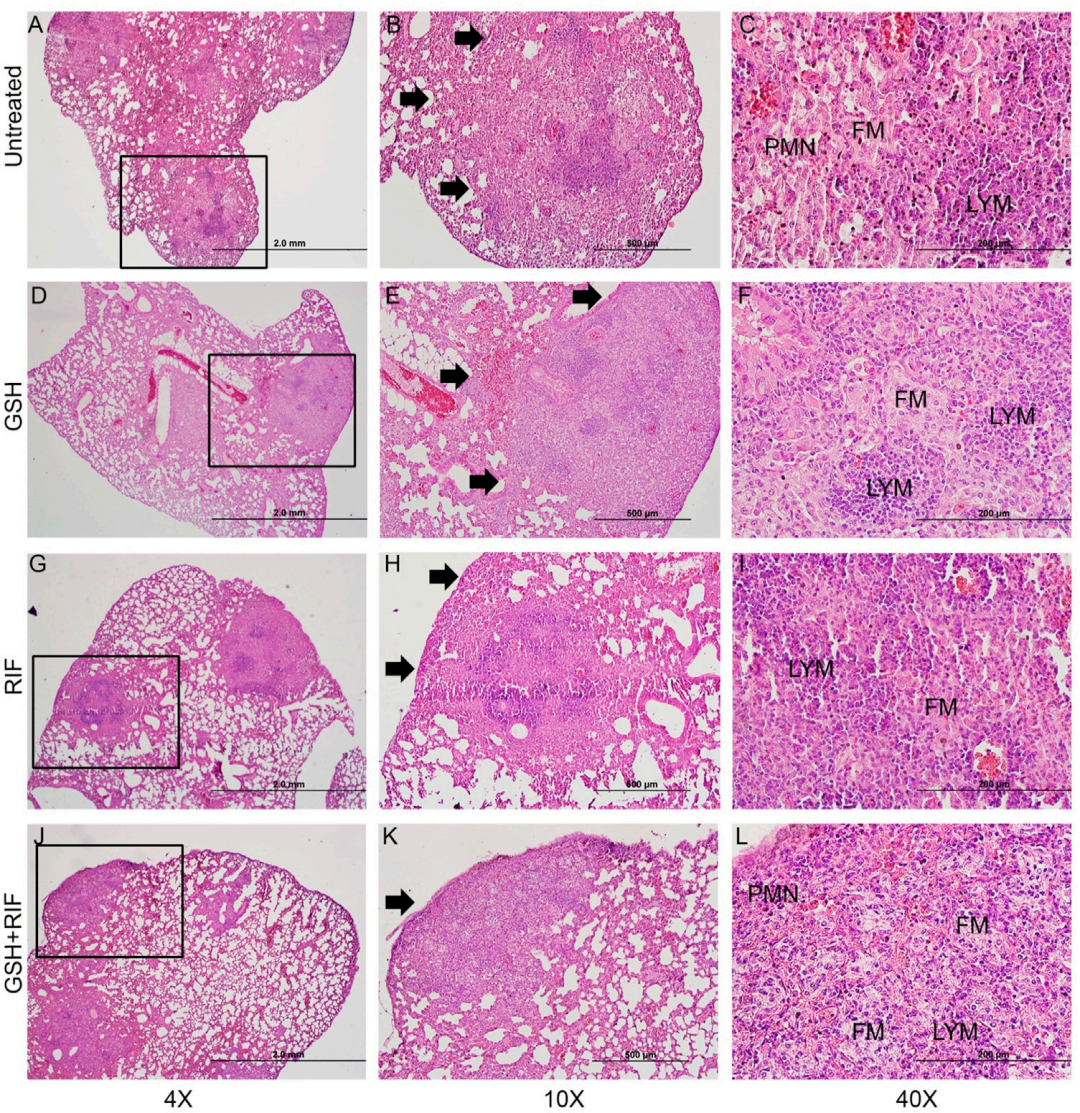
*Histology of M. tb infected db/db mice at 8 weeks post-infection.* Representative lung sections were collected from *M. tb* infected db/db mice with or without various treatments at 8-weeks post infection for H&E staining. Animals were either untreated **(A–C)** or treated with L-GSH (GSH; **(D–F)** or RIF **(G–I)** or a combination of L-GSH plus RIF (GSH + RIF; **(J–L)**. Images were photographed at 4X **(A,D,G,J)** or 10X **(B,E,H,K)** or 40x **(C, F, I, L)** magnifications. Boxes in 4X magnification represent the granulomatous region magnified in 10X. PMN- polymorphonuclear cells (neutrophils). FM-foamy macrophages; LYM-lymphocytes. The scale bar in 4X is 2 mm; 10X is 500 µm and 40X is 200 µm.

The lung histopathologic analysis at 8 weeks post-infection also revealed multiple, large, and poorly demarcated granulomatous lesions in all *M. tb* infected mice, irrespective of any treatments (Figures 10A–L). The size of granulomas at 8 weeks post-infection was larger and more in numbers compared to the lesions at 4 weeks post-infection in all groups of*M. tb* infected *db/db* mice. Like the 4 weeks lung lesions, abundant foamy macrophages were seen in the lung granulomas at 8 weeks post-infection, although no clear central necrosis was noted in any of the granulomas. In these granulomas, lymphocytes were clustered into multiple, small foci, particularly in the untreated (Figures 10A–C) and L-GSH-alone (Figures 10D–F) or RIF (Figures 10G–I) alone treated mice lungs. The PMN distribution was more prominent in the untreated and combined L-GSH + RIF treated mice lungs compared to the L-GSH-only treated animals. Although the granulomas were smaller in the lungs of mice treated with a combination of RIF + L-GSH (Figures 10J–L), compared to the other treatment groups at 8 weeks, they were larger in size than the lesions observed at 4-weeks post-infection. Further, prominent lymphocyte foci were rarely observed in the granulomas of RIF + L-GSH treated mice lungs compared to those animals treated with either L-GSH-alone or RIF alone.

Together, these observations suggest that L-GSH treatment does not exacerbate the granulomatous response, and treatment with RIF, an anti-TB drug, did not completely alleviate granuloma formation (4 weeks post-infection) or progression (8 weeks post-infection). Importantly, we observed that mice treated with a combination of RIF + L-GSH had the lowest extent of granulomatous response in the lungs among all the treatment groups at both 4 and 8 weeks post-infection.

### Diabetic Mice Infected With *M. Tb* Have Increased Levels of Plasma Glucose Than Wild-type Mice Infected With *M. Tb*

To reassess and explore plasma glucose levels among wild-type and *db/db* mice, a plasma glucose assay was performed. *db/db* mice infected with *M. tb* had significantly higher levels of plasma glucose when compared to *M. tb-*infected wild-type mice (Supplemental Figure S1). Additionally, plasma glucose levels were assessed among the *M. tb* infected db/db mice that were untreated, or treated with 40 mM L-GSH, RIF, or L-GSH and RIF. Our results showed that there is no significant difference in plasma glucose levels between any of the treatment groups in *db/db* mice at 4- and 8- weeks post-infection (Supplemental Figure S2).

## Discussion

It has been estimated that 30 million people in the United States are affected by type 2 diabetes mellitus (T2DM), with an additional 80 million in the early stage of pre-diabetes. T2DM is a systemic metabolic disease affecting a range of tissues and multiple organ systems. Because of this, T2DM is associated with co-morbidities, including vascular disease and increased susceptibility to *M. tb* infection. A 2017 report described that about 20% of people with TB in the United States were also afflicted with diabetes 1. T2DM increases the risk of developing active TB thirty-fold, thereby enhancing the mortality rate 1. The prevalence of TB worldwide has also been on the rise and is a public health concern.

Individuals with T2DM are immunocompromised and more susceptible to *M. tb* infection. It has been found that individuals with T2DM have reduced systemic GSH levels due to diminished levels of GSH-synthesizing enzymes. This phenomenon could be explained by the increased levels of TGF-β1, a cytokine known to decrease the expression of GCLC (Lagman et al., 2015). Along with increased TGF-β1 levels, individuals with T2DM have diminished levels of cytokines, such as TNF-α, IL-12, IFN-γ, and IL-2, that are responsible for controlling *M. tb*infection (Lagman et al., 2015). Previous research suggests that diminished GSH levels in T2DM lead to increased susceptibility to *M. tb* infection. Furthermore, it has been found that L-GSH supplementation reduced the burden of intracellular mycobacteria within *in vitro* granulomas generated with the peripheral blood mononuclear cells of T2DM subjects (To et al., 2021). L-GSH supplementation in T2DM has also been shown to increase the levels of Th1-response associated cytokines, such as IFN-γ, TNF-α, and IL-2, while diminishing the levels of immunosuppressive cytokines, such as IL-10 and IL-6 (To et al., 2021). Combining antioxidants with first-line antibiotics has been shown to be beneficial in augmenting bacterial clearance (Rao Muvva et al., 2021; To et al., 2021). One study on potential additive therapies to standard *M. tb* treatment has found that vitamin D along with phenylbutyrate (PBA) in combination with RIF inhibited mycobacterial intracellular growth*in vitro* (Rao Muvva et al., 2021). Additionally, N-Acetyl Cysteine (NAC), the precursor to GSH, when combined with RIF significantly lowered *M. tb* burden 15 days post-infectionwithin *in vitro* granulomas from PBMCs derived from healthy and T2DM individuals compared to RIF alone. (Teskey et al., 2018b). Exogenous GSH treatment has been shown to enhance antibiotic efficiency and efficacy in *Pseudomonas* infections *in vitro*. However, the synergistic effects of GSH and RIF during an *M. tb* infection have yet to be elucidated *in vivo* (Das et al., 2017). To expand upon previous research, we examined the effects of L-GSH supplementation in an *M. tb* infected diabetic (*db/db*) mouse model in conjunction with RIF treatment.

*M. tb* predominantly infects and multiplies in phagocytes, such as macrophages, inducing an inflammatory response, mainly mediated through the Th1 cells (de Martino et al., 2019). The Th1 cell’s response includes the release of cytokines, such as IFN-γ, which enhance macrophage effector functions that help in mounting the antimicrobial responses against *M. tb*. This also causes increased TNF-α release, which ultimately leads to the containment of *M. tb* in granulomas to prevent further spread of the infection (Flynn et al., 1995; Bean et al., 1999). However, an attribute of T2DM is systemic inflammation that causes immune dysregulation and promotes lipid peroxidation and oxidative stress, which can cause extensive tissue damage and *M. tb* dissemination (Pasupathi et al., 1970; Adarsh Pal Vig, 2012). L-GSH treatment significantly reduced levels of MDA, which further supports evidence that L-GSH functions as a free radical scavenger to neutralize harmful reactive oxygen species (ROS), thus limiting the severity of oxidative stress during *M. tb*infection (Lin and Flynn, 2010; Lagman et al., 2015; Gaucher et al., 2018). Our data also suggest a significantly elevated MDA level in diabetic mice treated with RIF alone, which can be explained by the mechanism of action of RIF. RIF binds to the beta subunit of *M. tb*’s DNA-dependent RNA polymerase to inhibit bacterial replication. Downstream signaling triggers the formation of the ROS, ·OH. These ROS are trapped by metal, DMPO, inherent to *M. tb,* which induces increased oxidative stress (Piccaro et al., 2014). When RIF is co-administered with L-GSH, oxidative stress levels, as measured via MDA, are significantly reduced, suggesting a potential for L-GSH to reverse the harmful oxidative effects of RIF while enhancing its bactericidal effects (Figure 2). Furthermore, the proinflammatory cytokine, IL-6, is implicated in the induction of oxidative stress. There was a significant decrease in IL-6 levels in the lungs of RIF with GSH treated diabetic mice infected with *M. tb* (Figure 6). These results further emphasize the benefit of L-GSH as an adjunctive treatment for *M. tb* infection by diminishing oxidative stress and enhancing RIF bactericidal activity.

IL-12 is a proinflammatory cytokine involved in the differentiation of naive CD4 T-cells to their Th1 subset, thus inducing the production of IFN-γ and IL-2. IFN-γ is a crucial cytokine that promotes the Th1 cell-mediated immune response by potentiating the effector functions of macrophages, NK cells, and CD8 T-cells. In the lungs of diabetic mice infected with *M. tb*, IL-12 and IFN-γ levels were significantly increased across all treatment groups and notably increased further in animals treated with a combination of RIF and L-GSH (Figure 3). The localized increase in the levels of IFN-γ in the combination treatment group is positively correlated with superior control of *M. tb* infection (Figure 3, Figure 7). The activated macrophages release TNF-α, which directs granuloma formation. The granuloma is composed of epithelioid histiocytes and multinucleated giant cells, which will contain *M. tb* and limit its dissemination (Mohan et al., 2001). L-GSH and RIF treatments both individually exhibit a significantly increased level of TNF-α, and this response is more robust when L-GSH and RIF are co-administered (Figure 6). Thus, the combination of L-GSH and RIF is positively correlated with an enhanced macrophage function and effective granulomatous response that efficiently controls *M. tb* proliferation in the lungs. Furthermore, treatment of *M. tb* infected diabetic mice with a combination of RIF and L-GSH reduced the size and number of granulomas (Figure 9). Combination treatment of RIF and L-GSH enhances standard RIF treatment by producing smaller and minimal granulomas, which can improve lung function by reducing the risk of permanent lung damage from *M. tb* infection.

Further evidence of GSH enhancement of the immune cell’s effector mechanisms against *M. tb* infection is exemplified by IL-17. Our data show that treatment with L-GSH alone and in the presence of RIF can increase IL-17levelsalthough, this increase did not reach significance. However, these results correlate with the increased bacterial control seen in mice treated with RIF and L-GSH compared to other treatment groups (Figure 10). IL-17 plays an important role in regulating mycobacterium-induced inflammation and establishing homeostasis (Khader and Cooper, 2008). These effects are critical to protecting tissues and organs from injury due to the excessive inflammation that may result from T2DM superimposed with *M. tb* infection. Furthering the evidence that supplementing RIF treatment with L-GSH can enhance immunoprotective mechanisms in *M. tb* infected diabetic mice, allowing for better control of the infection.

**FIGURE 11:**
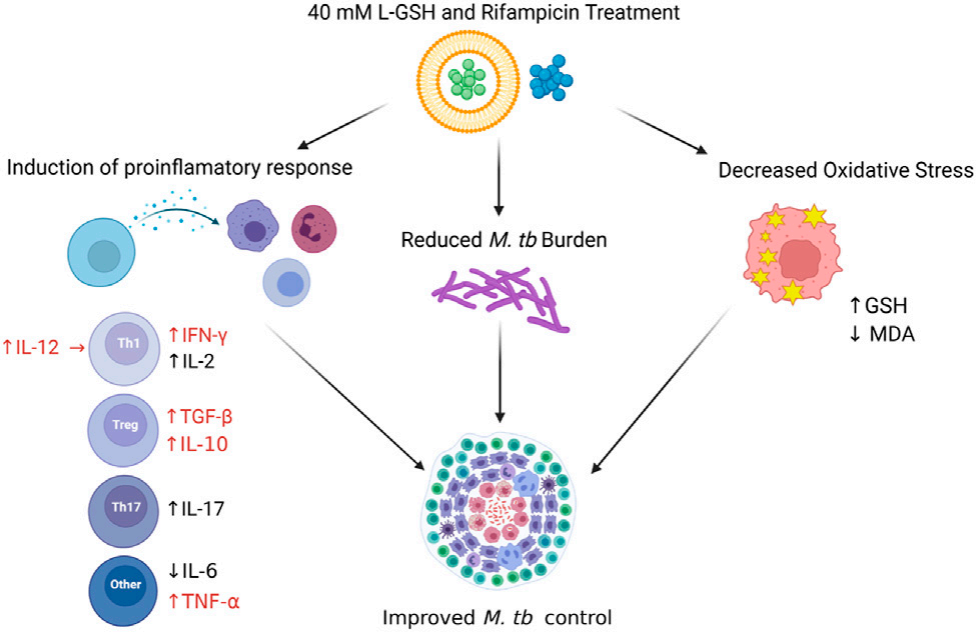
*Schema to show the implications of experimental findings in this study.* A combination treatment of RIF and L-GSH was associated with decreased oxidative stress, reduced *M. tb* burden, and induction of protective immune response in *M. tb* infected diabetic mice lung. RIF + L-GSH treatment resulted in increased levels of GSH and decreased levels of MDA, a by-product of lipid peroxidation, which indicates the decreased levels of oxidative stress in the lung. Treatment with RIF + L-GSH increased IL-12, IFN-γ, TGF**-**β**,** IL-10, IL-17, and TNF- α levels in the lungs. The number of *M. tb* CFUs was reduced in the lungs after RIF + L-GSH treatment. This figure was created using biorender.com.

Our data revealed a significant increase in the levels of IL-10 and TGF-β1 across the L-GSH and L-GSH plus RIF treatment groups. IL-10 and TGF-β1 exhibit anti-inflammatory effects and can regulate the immune response. T2DM is characterized by a proinflammatory state wherein there is an overproduction of cytokines which can damage the vasculature, interrupt hemodynamics, and increase the risk for infection (Casqueiro et al., 2012; Cruz et al., 2013). T2DM compromises the immune response, which allows *M. tb* to multiple and potentially disseminate to cause systemic infection (Jeon and Murray, 2008). L-GSH treatment, in the presence and absence of RIF, is positively associated with enhanced IL-10 and TGF-β1 activity which may elucidate a significant role of L-GSH in restoring homeostasis and regulating the immune response. The addition of L-GSH to RIF treatment can play an important role in regulating the immune response and thus allowing for better control of *M. tb* infection and reducing the potential of severe outcomes in diabetic individuals (Figure 11).

From our previous studies, we have implicated the role of GSH in controlling the levels of free radicals and oxidative damage and enhancing the immune response against *M. tb,* such that GSH depletion leads to immune dysregulation and more severe infection, which can be detrimental in immunocompromised individuals. In this study, we explored the additive effects of L-GSH in the presence and absence of rifampin in T2DM mice infected with *M. tb.* Our results suggest that L-GSH has direct antimycobacterial activity and can supplement treatment to reduce inflammation and oxidative stress, increase the levels of protective cytokines, and enhance the immune response to contain *M. tb* infection and limit its reactivation. This combination therapy against *M. tb* may be of significant importance in conditions such as T2DM and HIV, as it may provide more potent defenses against immune dysregulation and excess inflammation, which, if uncontrolled, can precipitate severe outcomes such as organ failure and systemic infection.

However, there are limitations to this study. Despite being widely considered as an important diabetic mouse model, *db/db* mice have relatively low-tolerance to rifampicin treatment as evident from the observed mortality when *M. tb*-infected mice were treated with 16 mg/kg rifampicin. We speculate that this mortality could be due to the inability of *db/db* mice to effectively metabolize rifampicin. We immediately resolved this challenge by administering *M. tb*-infected *db/db* mice with a lower dose of rifampicin (8 mg/kg) once every 2 days It is known that RIF induces its own metabolism in the host, however effects of dose on the extent of autoinduction in *db/db* mice have yet to be elucidated (Smythe et al., 2012). Due to the small number of mice used in this study, few data points were examined and no pharmacokinetics (PK) of RIF or L-GSH was performed in the diabetic C57BL *db/db* mice model. As this is a new model, data is still being generated and we intend to perform future studies to gain mechanistic insights on pharmacokinetics and the rifampicin-induced changes in the liver and other organs in both uninfected and *M. tb*-infected *db/db* mice. Future studies should perform *p*K/PD follow-up of the data with modeling to gain mechanistic details. Furthermore, we recognize our mouse model is different from the previously studied AKITA mouse model (Martens et al., 2007). The AKITA mouse model develops type 1 diabetes due to a mutation in the insulin 2 gene and has previously been established as a reliable model to study protective immunity against *M. tb* in the context of diabetes (Martens et al., 2007). Both the AKITA mouse model and ours have limited information on RIF pharmacokinetics and drug metabolism which necessitates further investigations to obtain a meaningful interpretation of these results (Franzblau et al., 2012). The findings from previous and future AKITA and *db/db* mouse model studies will be carefully analyzed to obtain a meaningful interpretation of how diabetes would contribute to the pathogenesis of tuberculosis and how L-GSH supplementation with RIF would improve the control of *M. tb* infection and pathogenesis. In the present study, the *M. tb* burden in the lungs, spleen and liver suggests that an association exists between the combined (L-GSH plus RIF) treatment and reduction in tissue bacillary load. However, to demonstrate the mechanistic basis for the relationship between L-GSH exposure with or without RIF and *M. tb* killing, additional studies, such as the kinetics of bacterial killing at different doses of L-GSH and RIF are needed. Similarly, the RIF dose used in our mice study was not consistent throughout the treatment duration. To avoid RIF-induced toxicity, we reduced the dose of this drug after 4 days of treatment (see methods). This change in drug dosing could affect the bacterial killing observed in the lung, liver and spleen. Therefore, the potential synergistic/additive effect of combination therapy with RIF and L-GSH on TB needs further evaluation of the time- and dose-response relationship between these compounds.

## Data Availability

The original contributions presented in the study are included in the article/Supplementary Material, further inquiries can be directed to the corresponding author.
