# Supplementary material for: L-GSH Supplementation in Conjunction With Rifampicin Augments the Treatment Response to Mycobacterium tuberculosis in a Diabetic Mouse Model
Source: Front Pharmacol. 2022 Jun 24;13:879729. doi: 10.3389/fphar.2022.879729 (PMC9263396; doi:10.3389/fphar.2022.879729)
Supplement: Supplementary file 1 [file DataSheet1.pdf]

Supplementary Figures

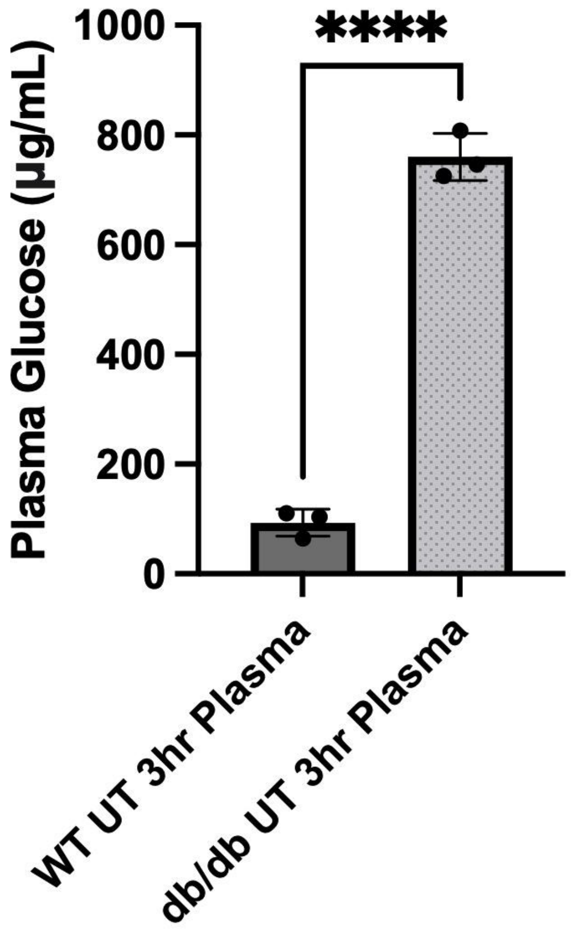

Supplementary Figure 1: Plasma Glucose levels in M. tb infected wild-type and diabetic mice. Plasma glucose levels were measured spectrophotometrically. Plasma was collected from WT and *db/db* mice infected with *M. tb*. GraphPad Prism Software 8 was utilized for statistical analysis. Values plotted represent the mean +/- standard deviation for each group with n=3 per group per time point. Data were analyzed using an unpaired T-test, with Welch corrections applied. p-values of < 0.05 (*), <0.01 (**), <0.005(***), <0.0001 (****) were considered significant.

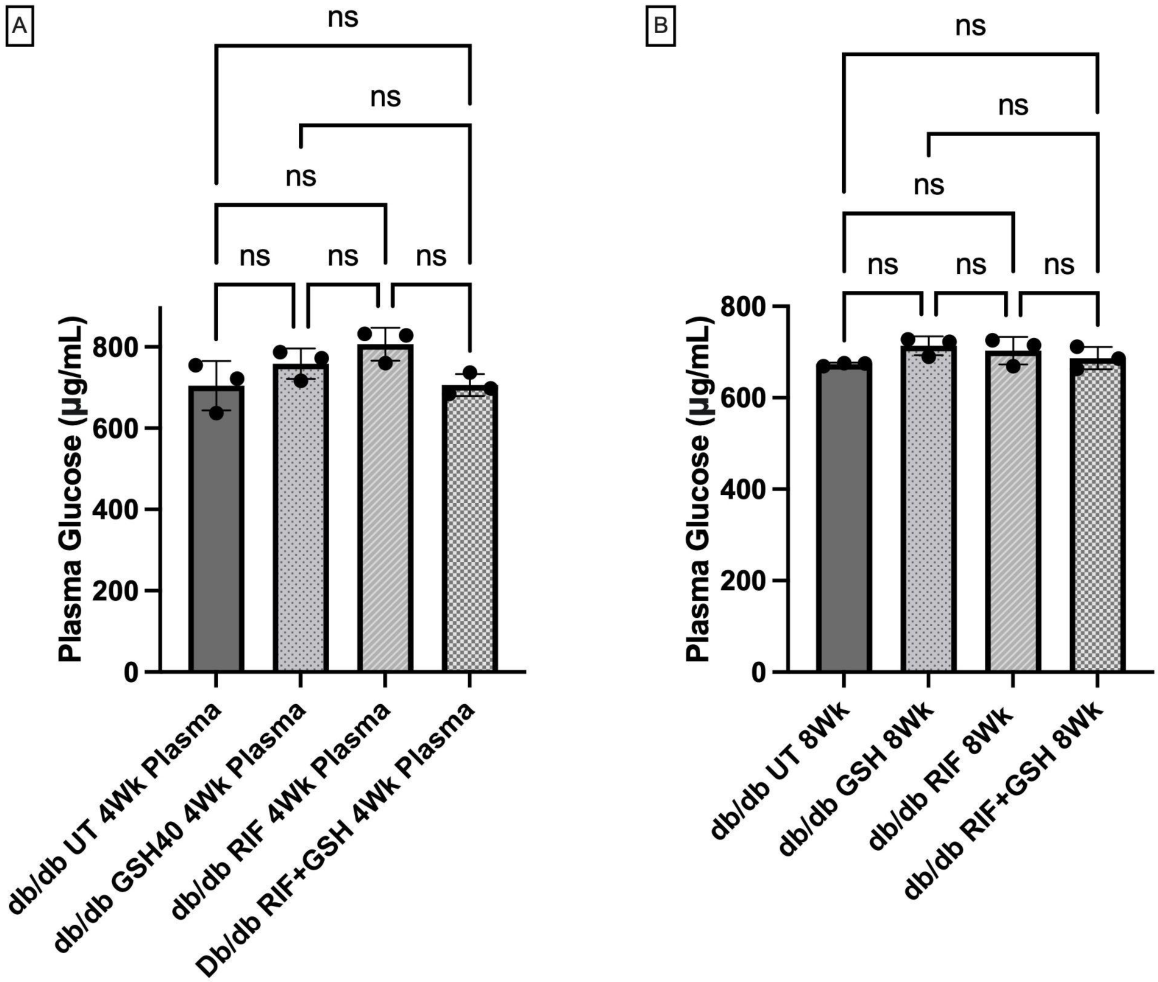

Supplementary Figure 2: Plasma Glucose Levels in untreated, 40mM L-GSH, RIF, or RIF + LGSH treated M. tb infected diabetic mice. Plasma glucose levels were measured spectrophotometrically. Plasma was collected from *M. tb* infected *db/db* mice that were untreated or treated with 40mM L-GSH, RIF, or RIF + L-GSH for 4-Weeks post-infection (A) and 8- weeks post-infection (B). GraphPad Prism Software 8 was utilized for statistical analysis. Values plotted represent the mean +/- standard deviation for each group with n=3 per group per time point. Data were analyzed using One-way ANOVA with Brown-Forsythe and Welch correction for unequal data dispersion.

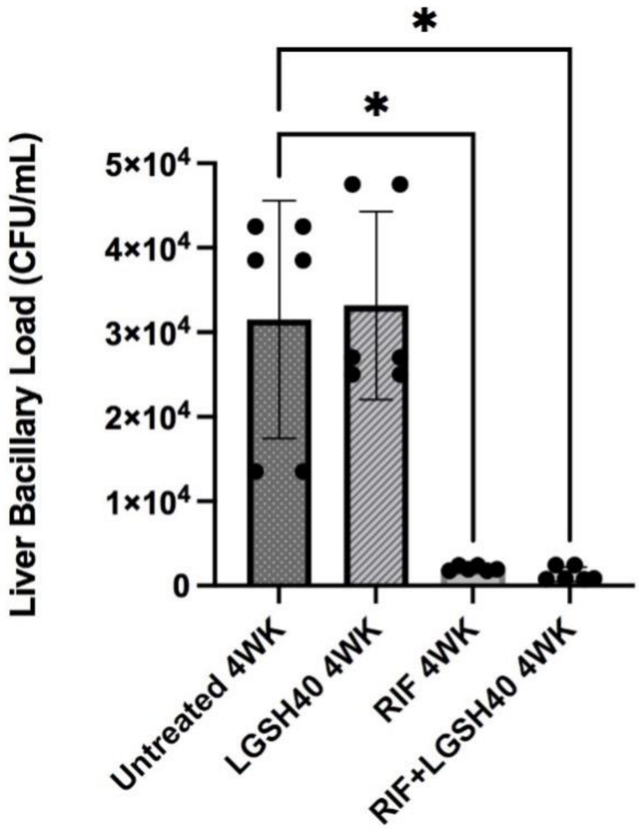

Supplementary Figure 3: M. tb burden in the liver of untreated, 40mM L-GSH, RIF, and RIF+ LGSH treated diabetic mice at 4-weeks post-infection. M. tb survival in the liver was quantified via CFU assays performed on liver homogenates of infected diabetic mice untreated or treated with 40 mM L-GSH, RIF, or RIF +L-GSH treatment at 4 weeks post-infection. GraphPad Prism Software 8 was utilized for statistical analysis. Values plotted represent the mean +/- standard deviation for each group with n=3 per group per time point. Data were analyzed as multiple groups using One-way ANOVA with Brown-Forsythe and Welch correction for unequal data dispersion. p-value of < 0.05 (*) was considered significant.
